# Speech disorders in patients with Tongue squamous cell carcinoma: A longitudinal observational study based on a questionnaire and acoustic analysis

**DOI:** 10.1186/s12903-023-02888-1

**Published:** 2023-04-01

**Authors:** Kaixin Guo, Yudong Xiao, Wei Deng, Guiyi Zhao, Jie Zhang, Yujie Liang, Le Yang, Guiqing Liao

**Affiliations:** 1 Department of Oral and Maxillofacial Surgery, Guanghua School of Stomatology, Hospital of Stomatology, Sun Yat-sen University, 56th Lingyuanxi Road, Guangzhou, Guangdong 510055 China; 2 Guangdong Provincial Key Laboratory of Stomatology, No.74, 2nd Zhongshan Road, Guangzhou, Guangdong 510080 China

**Keywords:** Tongue cancer, Speech disorders, Speech handicap index, Speech acoustics, Quality of life

## Abstract

**Background:**

Speech disorders are common dysfunctions in patients with tongue squamous cell carcinoma (TSCC) that can diminish their quality of life. There are few studies with multidimensional and longitudinal assessments of speech function in TSCC patients.

**Methods:**

This longitudinal observational study was conducted at the Hospital of Stomatology, Sun Yat-sen University, China, from January 2018 to March 2021. A cohort of 92 patients (53 males, age range: 24–77 years) diagnosed with TSCC participated in this study. Speech function was assessed from preoperatively to one year postoperatively using the Speech Handicap Index questionnaire and acoustic parameters. The risk factors for postoperative speech disorder were analyzed by a linear mixed-effects model. A t test or Mann‒Whitney U test was applied to analyze the differences in acoustic parameters under the influence of risk factors to determine the pathophysiological mechanisms of speech disorders in patients with TSCC.

**Results:**

The incidence of preoperative speech disorders was 58.7%, which increased up to 91.4% after surgery. Higher T stage (*P*＜0.001) and larger range of tongue resection (*P* = 0.002) were risk factors for postoperative speech disorders. Among the acoustic parameters, F2/i/decreased remarkably with higher T stage (*P* = 0.021) and larger range of tongue resection (*P* = 0.009), indicating restricted tongue movement in the anterior-posterior direction. The acoustic parameters analysis during the follow-up period showed that F1 and F2 were not significantly different of the patients with subtotal or total glossectomy over time.

**Conclusions:**

Speech disorders in TSCC patients is common and persistent. Less residual tongue volume led to worse speech-related QoL, indicating that surgically restoring the length of the tongue and strengthening tongue extension postoperatively may be important.

**Supplementary Information:**

The online version contains supplementary material available at 10.1186/s12903-023-02888-1.

## Background

Tongue squamous cell carcinoma (TSCC) is one of the most prevalent head and neck malignancies. Due to its increased incidence and decreased mortality [1], the quality of life (QoL) of patients with TSCC has become a significant issue [2, 3]. Dysphagia, psychiatric disorders and trismus seriously decrease QoL [4, 5]. However, speech is one of the most distressing problems for patients [6–8]. In previous studies, researchers confirmed that chemoradiotherapy and surgery affected patients’ speech intelligibility [9–11]. The tongue plays a significant role in speech due to its flexible mobility and strong muscles. The tumor itself, as well as the treatment, damages the integrity of the tongue structure and the coordination among pronunciation organs, greatly impairing speech function [12]. A longitudinal assessment of preoperative speech function and postoperative changes in patients diagnosed with TSCC helps accelerate the recovery of speech function. However, to the best of our knowledge, few studies have specifically focused on the longitudinal speech function of patients diagnosed with TSCC [13], particularly from a subjective and objective perspective.

At present, the commonly applied methods to evaluate speech function include perceptual evaluation [14], self-rating [3] and instrumental evaluation, such as magnetic resonance imaging (MRI) of the articulator [15–18] and lingual ultrasound imaging [19, 20]. Among them, acoustic parameters scratched and filtered from audio have been increasingly used because of their ability to reflect the vocal tract and vocal folds, providing objective and quantitative descriptions of speech disorders [21, 22]. It has been proven that the assessment of speech spectral characteristics can show postoperative changes in speech function in patients with oral squamous cell carcinoma [23]. The first formant frequency (F1) and second formant frequency (F2) are two relevant acoustic parameters that are primarily determined by the tongue position, reflecting the production of vowels. They are related to tongue elevation and anterior-posterior movement, respectively [22]. Based on F1 and F2, more comprehensive acoustic parameters have been proposed. One of the most often utilized acoustic characteristics is the vowel space area (VSA) [24, 25]. It has been proven that the smaller the VSA, the worse the speech intelligibility [26, 27]. Some studies have found its clinical relationship with many speech-related disorders, such as Parkinson’s disease (PD) [28] and multiple sclerosis (MS) [29]. In addition, the formant centralization ratio (FCR) was first proposed by Sapir et al. [30]. It is a comprehensive parameter of dynamic connotations, better reflecting articulation status [31]. The VSA and the FCR have been proven to appropriately describe the quality of vowel articulation and are associated with intelligibility [32]./a/,/i/,/u// are three basic vowels, and the graph of a vowel triangle composed of them in the F1-F2 plane can visually display the range of tongue movement. However, acoustic analysis can be affected by the cognitive level, cultural differences and the dialects of the speaker. In addition, speech disorders have subjective effects on patients, and in a previous study, investigators reported that an acoustic analysis was not correlated with questionnaires, so it cannot assess speech function independently [33]. Questionnaires can be an auxiliary method to assess speech function, reflecting the complaints of patients with different demands. In 2008, Rinkel et al. designed a speech-specific questionnaire, the Speech Handicap Index (SHI) [34], which has been proven to be a valid and accurate method for evaluating the speech-related QoL of patients diagnosed with oral cancer. We proposed that acoustic parameters supplemented with speech-specific questionnaires can provide a more comprehensive assessment of speech disorders in patients with TSCC.

This study revealed the variation in speech function in TSCC patients from preoperatively to one year postoperatively. In this study, important postoperative factors in speech disorders were discovered by using subjective and objective assessment methods. The authors aimed to discover the pathophysiological mechanisms suggested by acoustic parameters to provide theoretical support for postoperative speech function rehabilitation.

## Methods

### Subjects

**Fig. 1:**
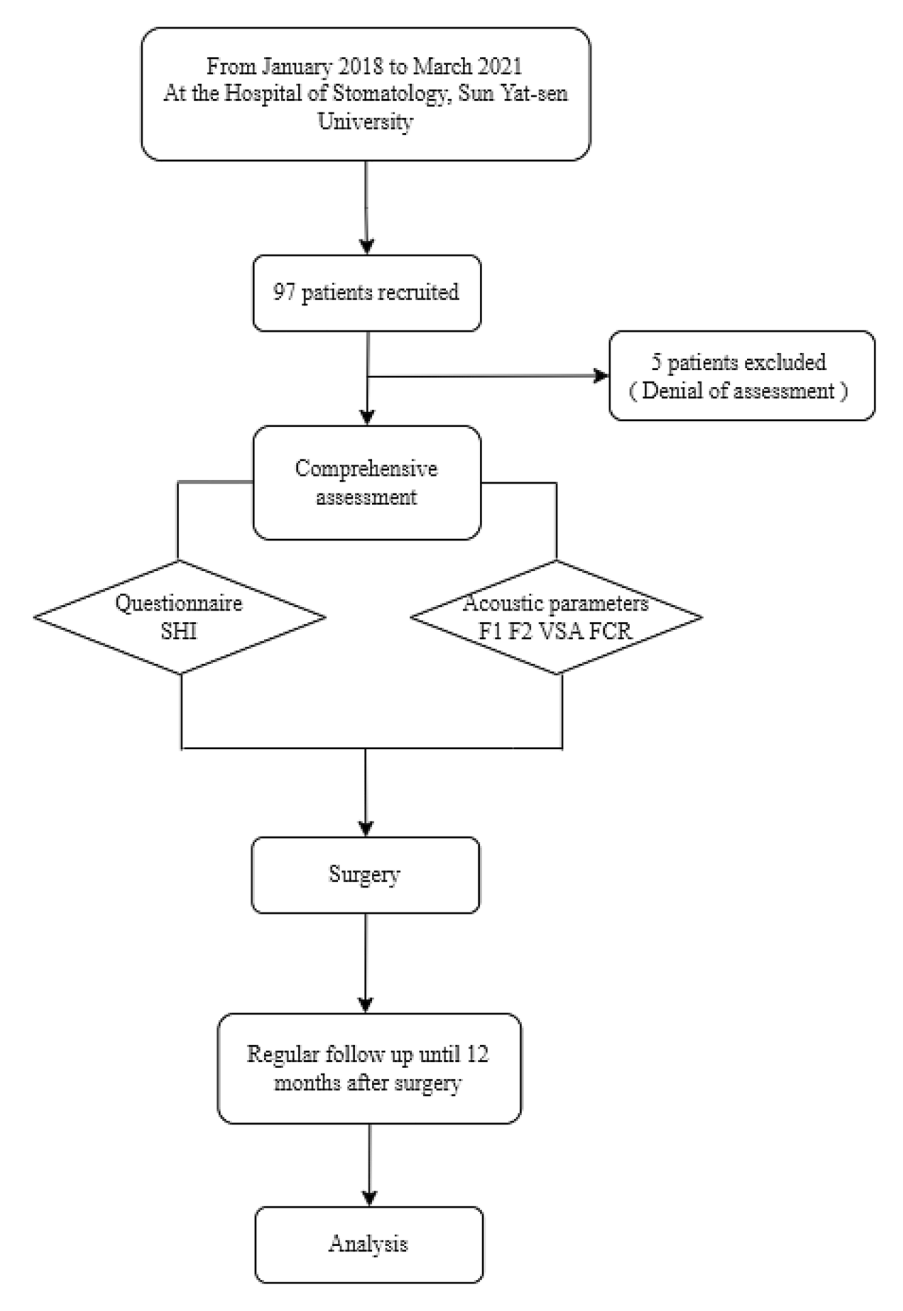
Study design flowchart

We conducted this study at the Department of Oral and Maxillofacial Surgery, Hospital of Stomatology, Sun Yat-sen University, China, from January 2018 to March 2021 (Fig. 1). To ensure the validity of the experiment, participants were required to (a) be older than 18 years but younger than 80 years, (b) have squamous cell carcinoma of the tongue with histological confirmation, (c) have undergone tumor resection, (d) be fluent in Mandarin for daily communication, and (e) have no neurological diseases. Individuals were excluded if (a) they had no complete speech evaluation before surgery or (b) they had other diseases affecting speech, such as nasal obstruction and maxillary defects. Data were obtained longitudinally at several time points: within one week before surgery and one month, one to three months, three to six months and twelve months after surgery. The sociodemographic information and disease variables should be collected before the first speech assessment. The surgery-related details, such as tracheotomy and reconstruction methods, were extracted from the medical record system.

### Assessment of speech-related QoL

The 30-item Speech Handicap Index (SHI) is a speech-specific questionnaire that focuses on the patients’ speech-related QoL. Response scores range from 0 to 4, with 0 indicating “never”, 1 indicating “almost never”, 2 indicating “sometimes”, 3 indicating “almost always” and 4 indicating “always”. The overall score is between 0 and 120. A higher score indicates a worsening of the speech disorder. As the cutoff value, a score of 6 indicates the presence of a speech disorder and deterioration in speech-related QoL. To further stratify patients’ speech problems, the SHI questionnaire is divided into two subscales, psychosocial function and speech function, both of which contain 14 items.

### Recording and extraction of acoustic parameters

The entire procedure was conducted in a low-noise environment, and all speech samples were collected in a quiet environment at the outpatient department of Oral and Maxillofacial Surgery at the Hospital of Stomatology, Sun Yat-sen University. The speech samples were recorded with a Samson CO3U USB Multipattern Condenser Microphone and speech recording software called Field Phon. Researchers were trained on how to operate the devices. The sound waveform was set to sample at 22,050 Hz with a minimum of 16-bit resolution. Patients were required to pronounce the /a/, /i/, /u/ at a comfortable volume and pitch two to three times for at least two seconds. After audio collection, we used Praat (version 6.0.49, 2018) for noise reduction, manual labeling, and segmentation. F1 and F2 were extracted by internal scripts and denoted as F1/a/, F2/a/, F1/i/, F2/i/, F1/u/, and F2/u/ for/a/, /i/, and/u/, respectively. Furthermore, the FCR and VSA were computed (see Eqs. 1–2). The VSA reflected the range of tongue motion in the F1-F2 plane. The vertical and horizontal axes are F1 and F2, respectively.

1 $$FCR=\frac{\text{ F2/a/+ F2/u/+F1/i/+F1/u/}}{\text{ F2/i/+F1/a/}}$$

2 $$VCA=0.5\times \left|\begin{array}{cc}\text{F1/a/}& \begin{array}{cc}\text{F2/a/}& 1\end{array}\\ \begin{array}{c}\text{F1/i/}\\ \text{F1/u/}\end{array}& \begin{array}{c}\begin{array}{cc}\text{F2/i/}& 1\end{array}\\ \begin{array}{cc}\text{F2/u/}& 1\end{array}\end{array}\end{array}\right|$$

### Statistical analysis

SPSS version 25.0 and GraphPad Prism 8.0.2 were used for the statistical analyses. A linear mixed-effects model analysis was applied to identify the independent postoperative risk factors for a speech disorder. In univariate analysis, we compared continuous variables with t tests, Mann‒Whitney U tests or one-way analysis of variance (one-way ANOVA) where appropriate. One-way ANOVA was also applied in the analysis of acoustic parameters during the follow-up period. A two-sided, *P* value of 0.05 or less indicated statistical significance.

## Results

### Subjects

Ninety-seven patients participated in this study, and 5 were excluded for refusing postoperative speech assessment. The cohort of 92 patients consisted of 53 males and 39 females, ranging in age from 24 to 77 years old, with a mean age of 49.45 years. T stage was categorized into T_1 − 2_ (n = 60) and T_3 − 4_ (n = 32) for statistical analysis. N stage was divided into N_0_ (n = 60) and N_+_ (n = 32). Among all the participants, approximately 42.4% (n = 39) were diagnosed with comorbidities, including but not limited to diabetes and high blood pressure. Approximately 28.3% of patients received chemoradiotherapy postoperatively. The detailed information is delineated in Table 1.

**Table 1:** Patients’ characteristics

Characteristics	No.(%)
Gender	
Male	53 (57.6)
Female	39 (42.4)
Age	
＜50	45 (48.9)
≥ 50	47 (51.1)
Smoking	
Yes	39 (42.4)
No	53 (57.6)
Excessive drinking	
Yes	16 (17.4)
No	76 (82.6)
Habitual betel nut chewing	
Yes	13 (14.1)
No	79 (85.9)
Pain	
Yes	68 (73.9)
No	24 (26.1)
Restricted tongue movement	
Yes	64 (69.6)
No	28 (30.4)
Comorbidity	
Yes	39 (42.4)
No	53 (57.6)
T stage	
T_1_ or T_2_	60 (65.2)
T_3_ or T_4_	32 (34.8)
N stage	
N_0_	60 (65.2)
N_+_	32 (34.8)
Tumor sublocation	
Belly of tongue	28 (30.4)
Edge of tongue	44 (47.8)
Others	20 (21.7)
Tracheotomy	
Yes	53 (57.6)
No	39 (42.4)
Reconstruction method	
No Free flap Pedicle flap	33 (34.4) 42 (43.8) 17 (18.5)
Range of tongue resection Partial glossectomy Hemiglossectomy Subtotal/total glossectomy	33 (35.9) 28 (30.4) 31 (33.7)
Postoperative chemoradiotherapy Yes No	26 (28.3) 66 (71.7)

### SHI and acoustic parameters before and after surgery

**Fig. 2:**
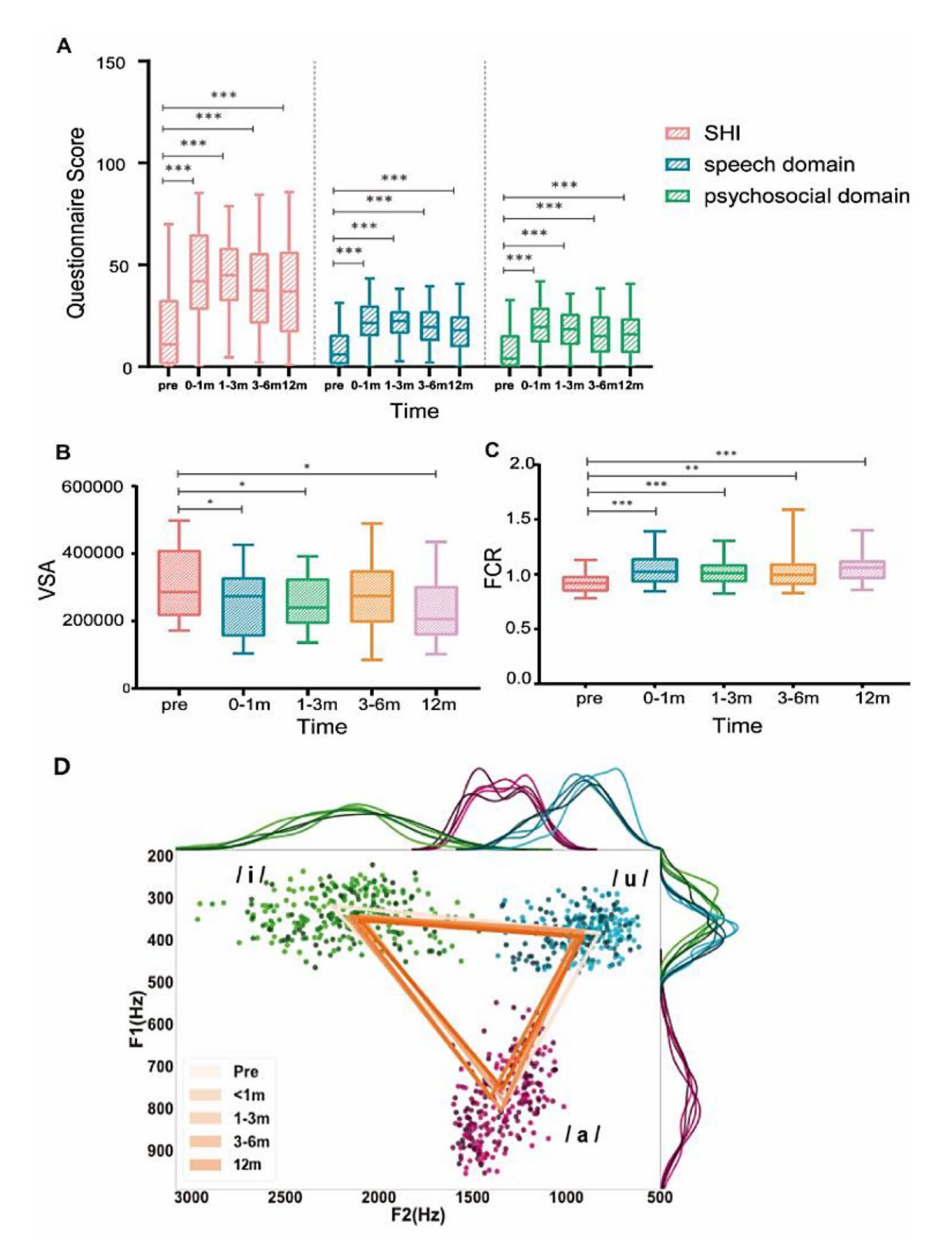
Variations of SHI and acoustic parameters from preoperative to one year postoperative. (A) The variation of SHI (total, speech, and psychosocial domains), (B) the variation of acoustic feature VSA, (C) the variation of acoustic feature FCR. (D) Triangular vowel space. The dots painted with green, pink and blue represent the what place the tongue occupied in the mouth when pronouncing /i/, /a/ and /u/ respectively. And their corresponding F1 and F2 are shown as lines above and to the right

The incidence of speech disorders (SHI ≥ 6) was 58.7% preoperatively, increasing to 91.2% after surgery. Speech-related QoL was impaired significantly, with SHI scores increasing from 20.86 to 45.21 after surgery (*P* < 0.05). Furthermore, speech function did not significantly improve within a year even though patients’ self-evaluation reports showed a better trend but without statistical significance (Fig. 2A). The acoustic parameter results showed that the FCR was higher postoperatively, and the VSA was lower postoperatively (Fig. 2B, C), both of which indicating a reduced range of tongue movement. A vowel triangle graph composed of/a, i, u/in the F1-F2 plane displayed the changes in the range of tongue motion at different periods (Fig. 2D). The VSA was negatively correlated with the SHI score (r=-0.409, P < 0.001), which indicated that less range of tongue movement led to worse speech-related QoL.

### The risk factors for postoperative speech disorder and the pathophysiological mechanisms suggested by acoustic parameters

A linear mixed-effects model (LMM) was applied to determine the independent risk factors for postoperative speech disorder. The results are listed in Table 2. Range of tongue resection (*P* = 0.002) and T stage (P < 0.001) had the main effect (Figure S1). Patients with a larger range of tongue resection or higher T stage had worse speech-related QoL.

**Table 2:** Linear mixed-effected model analysis of independent risk factors of postoperative speech disorder

Dependents	SHI	
	*P*-value	*F*
Tracheotomy	0.334	0.945
Time	0.036*	3.166
Tracheotomy × Time	0.311	1.206
Reconstruction	0.365	1.314
Time	0.026*	3.475
Reconstruction × Time	0.365	1.103
Range of tongue resection	0.002*	6.511
Time	0.069	2.577
Range of tongue resection × Time	0.301	1.222
Postoperative chemoradiotherapy	0.519	9.871
Time	0.002*	0.7574
Postoperative chemoradiotherapy ×Time	0.728	0.4354
T stage	<0.001*	15.53
Time	0.325	1.158
T stage × Time	0.340	1.131
N stage	0.165	1.964
Time	0.101	2.217
N stage × Time	0.936	0.140

**Fig. 3:**
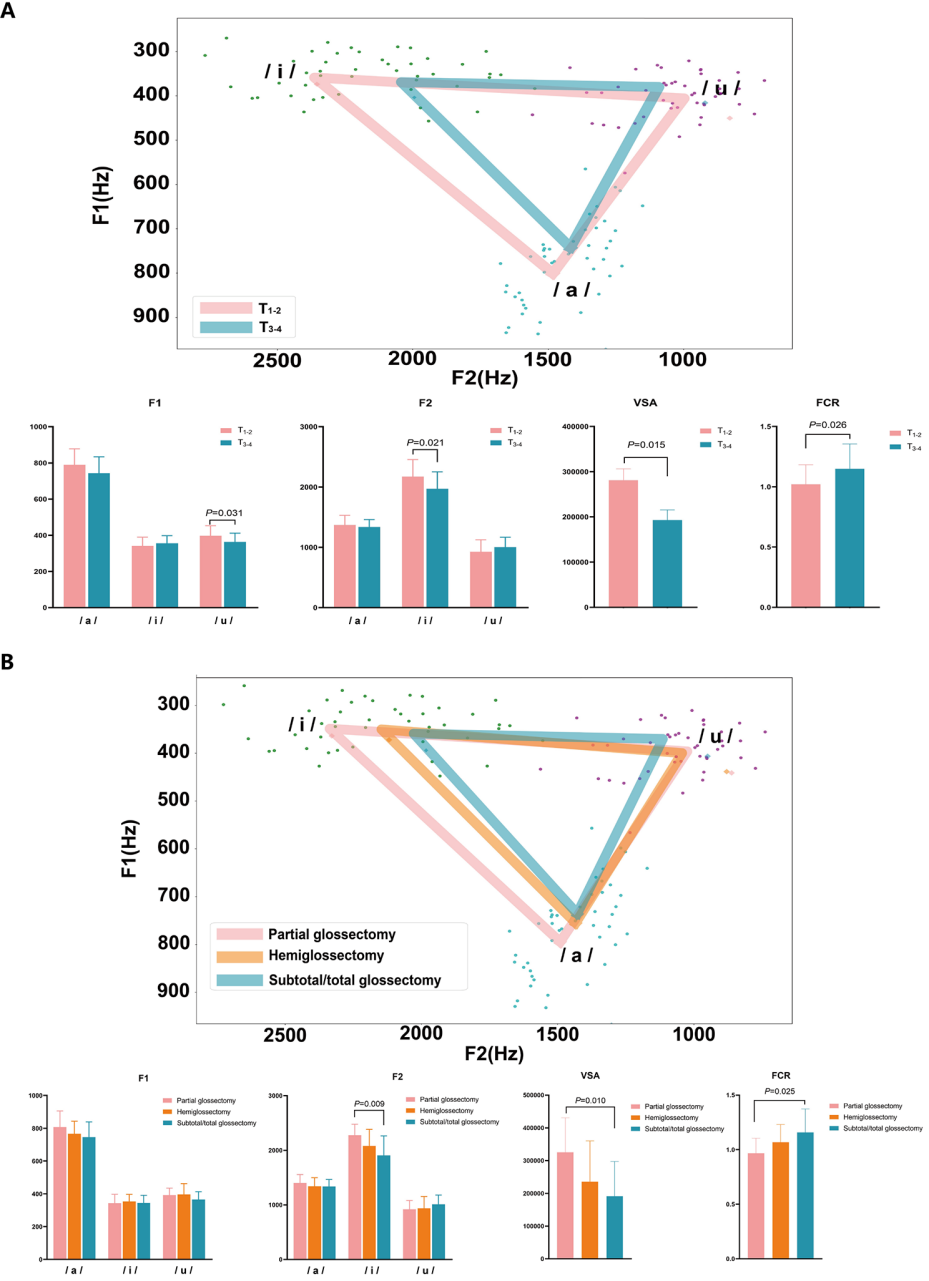
Difference of the acoustic features and vowel space area in F1-F2 plane caused by the independent risk factors. (A) T stage, (B) range of tongue resection

After screening out the TSCC patients with postoperative speech disorder (SHI ≥ 6), acoustic features were assessed three months after surgery to analyze the possible influence of the acoustic mechanisms. Patients were divided into groups according to T stage and range of tongue resection. For the T stage, the vowel triangle in the F1-F2 plane showed that the range of tongue motion was smaller in T_3 − 4_ (Fig. 3A). According to the statistics, the VSA in group T_1 − 2_ was smaller, and the FCR was larger. In other words, the higher the patient’s T stage, the more restricted the range of tongue motion. Among them, F1/u/and F2/i/played significant roles. Group T_3 − 4_ had smaller F1/u/and F2/i/. Mapping to the geometric triangle, the distance between/i/ and/u/ was shorter in group T_3 − 4_, which meant restricted tongue movement in the sagittal direction.

One-way ANOVA among the three classes of range of tongue resection revealed that the VSA increased (*P* = 0.010) and the FCR decreased (*P* = 0.025) as the extent of tongue resection increased (Fig. 3B). Patients who underwent partial glossectomy had a significantly higher F2 during articulation of the vowel/i/, indicating a more restricted range of tongue movement in the sagittal plane during vowel pronunciation as the residual tongue volume decreased.

### Trends of acoustic parameters under the influence of independent risk factors

**Fig. 4:**
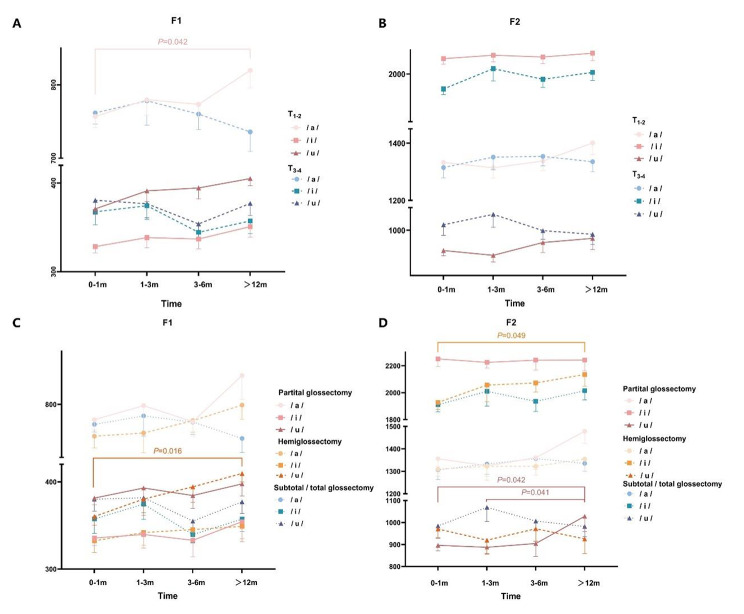
Variations of F1 and F2 of three vowels /a/, /i/, /u/ under the influence of T stage and range of tongue resection. (A, B) T stage, (C, D) range of tongue resection

The alteration of F1 and F2 under the influence of residual tongue volume over time was clarified by one-way ANOVA. (Fig. 4). For patients with T_1 − 2_, F1/a/increased gradually after surgery (*P* = 0.042), which meant that the height of the tongue elevation increased. For the range of tongue resection, F1/u/(*P* = 0.016) and F2/i/markedly increased (*P* = 0.049) in the hemiglossectomy group; in other words, the movement of the tongue in the anterior and posterior directions increased over time. In the partial glossectomy group, F2/u/began to increase at three months postoperatively (*P* = 0.042), which indicated anterior displacement of the tongue’s backward extension. The F1 and F2 of the three vowels did not change in the subtotal or total tongue resection group within one year after surgery.

## Discussion

This study elucidated the high incidence of speech disorder development before and after surgery, showing the variation in acoustic characteristics before surgery and one year after surgery. After the speech assessment, we explored whether the residual tongue volume was a risk factor for postoperative speech disorder development and tried to determine the pathophysiological mechanisms suggested by acoustic parameters.

Our work assessed speech function through questionnaires and acoustic analysis. Currently, widely used QoL questionnaires of OSCC include the European Organization for Research and Treatment of Cancer (EORTC) QLQs [22, 35] and the Functional Assessment of Cancer Therapy (FACT) scale [2]. However, their focus on speech is minimal, which cannot accurately reflect the patient’s speech function. The selected questionnaire was the SHI, which was specifically designed for patients diagnosed with oral cancer to obtain a better understanding of their speech function. The Chinese version has been translated and proven to be reliable and valid [36]. In the acoustic analysis, F1 and F2 are two relevant parameters that can reflect the vocal tract of vowel pronunciation. Mapping in the geometric triangle can better visualize the variation in tongue motion position. The VSA and the FCR are two comprehensive parameters reflecting the range of tongue movement [35]. Currently, the demand for noninvasive screening tests, such as tissue fluorescence imaging [37], salivary metabolomics [38], and serological markers [39], has greatly increased [40]. Our previous study found that the FCR could be a potential acoustic marker for detecting speech disorders in patients with TSCC [41]. This study confirmed that sufficient long-term data after surgery could show that the T stage could affect the FCR. After collecting and analyzing the full-cycle acoustic parameters of TSCC patients to find the varying pattern, an acoustic analysis has great potential as a novel and convenient noninvasive tumor screening method. It has been effectively used for some diseases [42–44].

The incidence of preoperative speech disorders in patients with TSCC was up to 58.7%, indicating that preoperative speech impairment in TSCC patients also needs to be taken seriously. In previous studies, researchers have noticed that the speech intelligibility is worse in TSCC patients than in the normal population before surgery [45, 46]. However, they did not find a high incidence of preoperative speech disorders. Pain and restricted tongue movement caused by tumor invasion may be the underlying factors [32]. Focusing on patients’ speech impairment as soon as possible may help to enhance communication and build a solid relationship between doctors and patients. Timely dissemination of information allows patients to psychologically prepare for future situations, thus helping the treatment proceed smoothly.

Analysis of the LMM revealed that T stage and range of tongue resection were independent risk factors influencing speech function. Patients with a higher T stage or a larger range of tongue resection have a higher mean SHI score and a smaller VSA. In other words, speech disorders were more serious in patients with less residual tongue volume, which is consistent with previous studies [47–50]. Among them, the decrease in F2/i/played the most significant role, indicating that patients’ impaired speech function was mainly caused by limited movement in the anterior-posterior dimension. This result was consistent with the study by Whitehill TL [35] and Narayanan SS [48]. The results of this study can be used to assure surgeons that restoring tongue length can help resolve speech function impairment in TSCC patients undergoing mass resection, primary closure or flap reconstruction. For SLPs, strengthening tongue extension in postoperative speech rehabilitation may help improve the speech function of TSCC patients.

The analysis of the postoperative follow-up period showed that F1 and F2 of patients who underwent subtotal or total tongue resection had no obvious changes within one year after surgery. Therefore, timely postoperative speech rehabilitation is very important for this group of patients. Patients with smaller tumors and defects after surgery had obvious changes in F1. Tongue elevation can be increased in patients with early-stage tumors by adjusting the muscles of the mouth to allow better contact with the palate under natural recovery. We found that F2/i/increased gradually in patients with hemiglossectomy, suggesting that increasing the length of tongue extension in patients with moderate defects can achieve better articulation because the position of the tongue is more precise. This result is consistent with a previous study in which patients undergoing glossectomy developed irregular muscle movement patterns [51].

The limitation of this study was its retrospective nature. Additionally, we only applied two methods to assess speech function without perceptual evaluation because of the small sample size. Nevertheless, this study focused on the preoperative and postoperative speech disorders of TSCC patients. We found that T stage and range of tongue resection were risk factors for speech disorder development. The reduction in tongue movement in the sagittal direction may be the main reason for persistent postoperative deterioration of speech function. Therefore, postoperative speech rehabilitation should be initiated as early as possible.

## Conclusions

In summary, this study revealed the high prevalence of perioperative speech disorders in TSCC patients. The length of tongue extension influenced speech function, suggesting that surgical restoration of tongue length and increasing the tongue extension postoperatively may help to improve speech-related QoL.

## Electronic supplementary material

Below is the link to the electronic supplementary material.

Supplementary Figure S1

## Data Availability

The datasets used and/or analysed during the current study are available from the corresponding author on reasonable request.

## Abbreviations

TSCC: Tongue squamous cell carcinoma
QoL: Quality of life
F1: First formant frequency
F2: Second formant frequency
SHI: Speech handicap index
VSA: Vowel space area
FCR: Formant centralization ratio
