# Supplementary figures and images for: Speech disorders in patients with Tongue squamous cell carcinoma: A longitudinal observational study based on a questionnaire and acoustic analysis
Source: BMC Oral Health. 2023 Apr 1;23:192. doi: 10.1186/s12903-023-02888-1 (PMC10068158; doi:10.1186/s12903-023-02888-1)

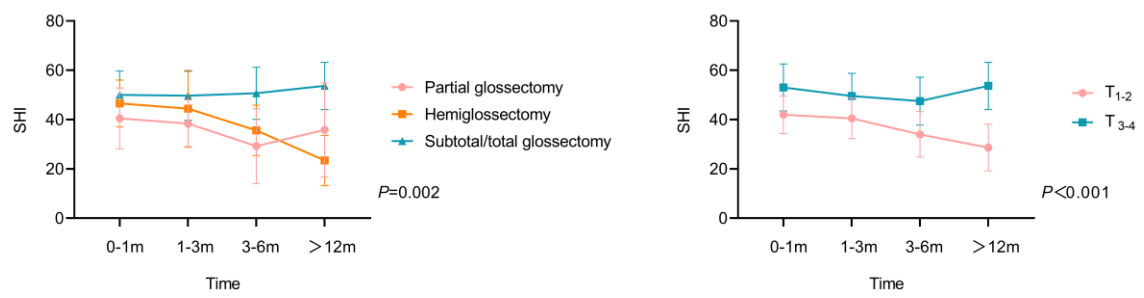

**Figure S1.** Linear mixed-effected model of postoperative SHI.

Supplement: Supplementary file 1 — Supplementary Figure S1 [file 12903_2023_2888_MOESM1_ESM.pdf]
